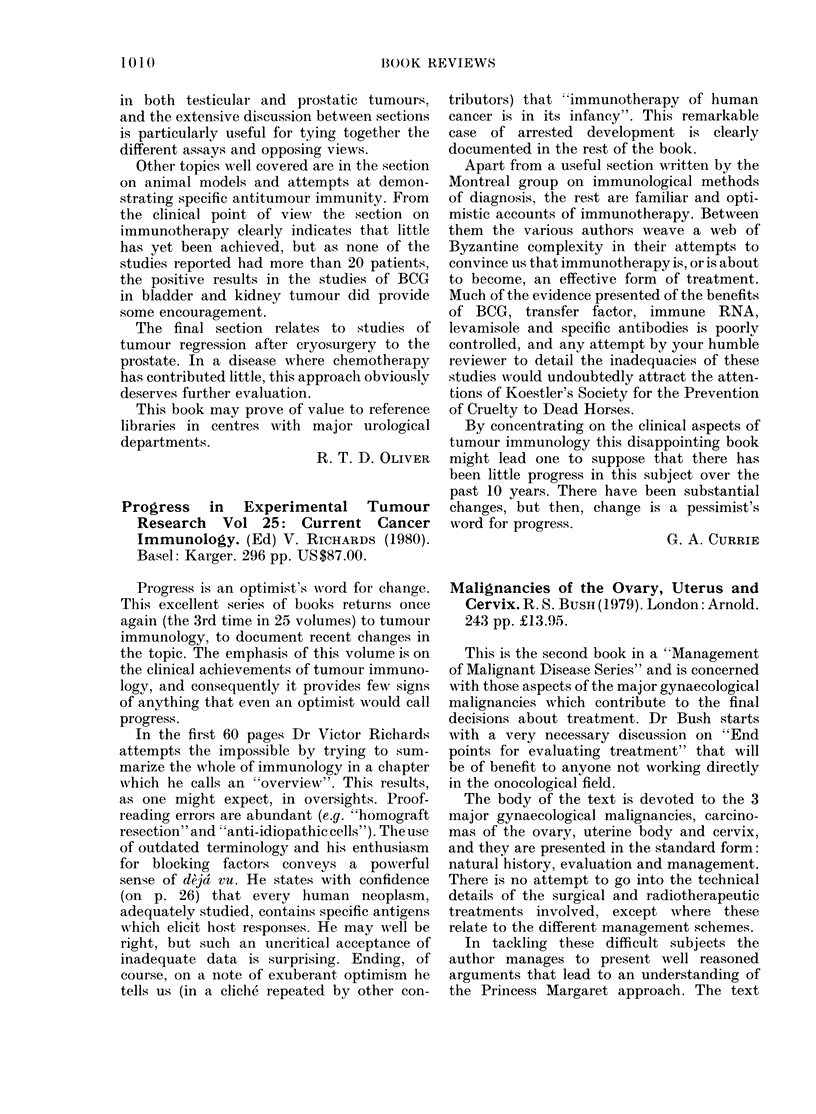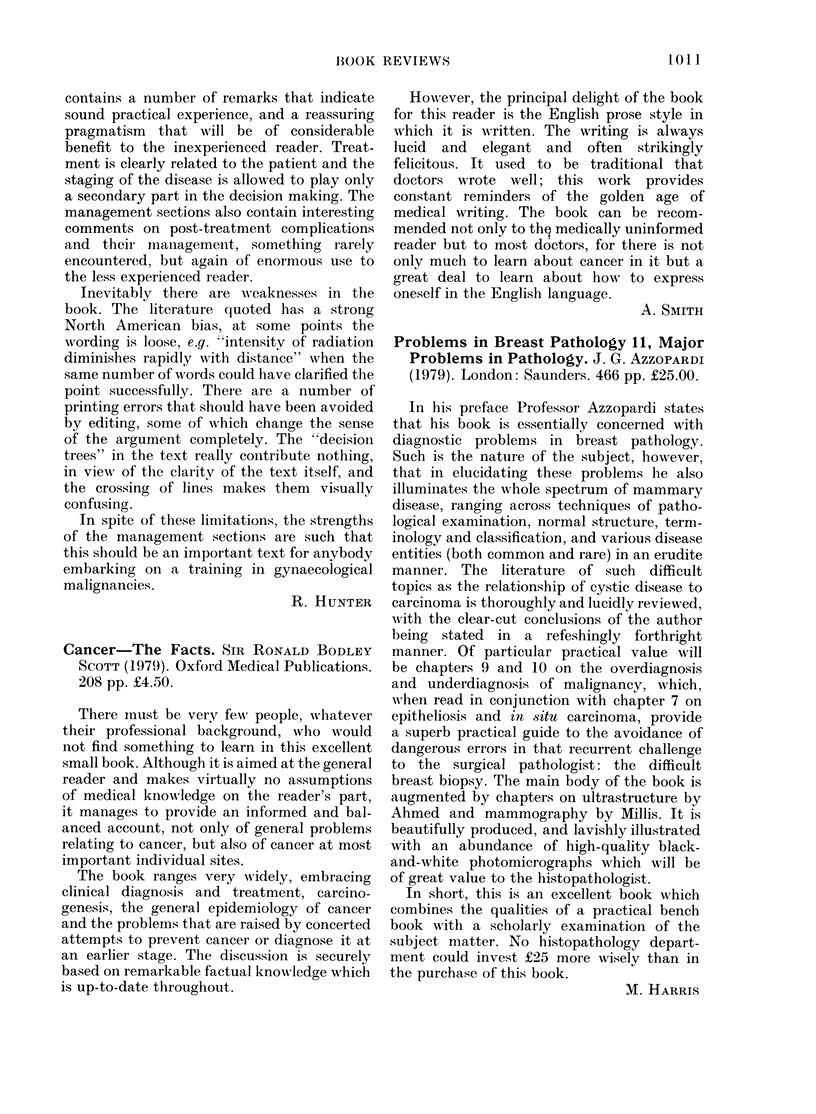# Malignancies of the Ovary, Uterus and Cervix

**Published:** 1980-06

**Authors:** R. Hunter


**Malignancies of the Ovary, Uterus and Cervix.** R. S. Bush (1979). London: Arnold. 243 pp. £13.95.

This is the second book in a "Management of Malignant Disease Series" and is concerned with those aspects of the major gynaecological malignancies which contribute to the final decisions about treatment. Dr Bush starts with a very necessary discussion on "End points for evaluating treatment" that will be of benefit to anyone not working directly in the onocological field.

The body of the text is devoted to the 3 major gynaecological malignancies, carcinomas of the ovary, uterine body and cervix, and they are presented in the standard form: natural history, evaluation and management. There is no attempt to go into the technical details of the surgical and radiotherapeutic treatments involved, except where these relate to the different management schemes.

In tackling these difficult subjects the author manages to present well reasoned arguments that lead to an understanding of the Princess Margaret approach. The text contains a number of remarks that indicate sound practical experience, and a reassuring pragmatism that will be of considerable benefit to the inexperienced reader. Treatment is clearly related to the patient and the staging of the disease is allowed to play only a secondary part in the decision making. The management sections also contain interesting comments on post-treatment complications and their management, something rarely encountered, but again of enormous use to the less experienced reader.

Inevitably there are weaknesses in the book. The literature quoted has a strong North American bias, at some points the wording is loose, *e.g.* "intensity of radiation diminishes rapidly with distance" when the same number of words could have clarified the point successfully. There are a number of printing errors that should have been avoided by editing, some of which change the sense of the argument completely. The "decision trees" in the text really contribute nothing, in view of the clarity of the text itself, and the crossing of lines makes them visually confusing.

In spite of these limitations, the strengths of the management sections are such that this should be an important text for anybody embarking on a training in gynaecological malignancies.

R. HUNTER